# Early adolescent pregnancy increases risk of incident HIV infection in the Eastern Cape, South Africa: a longitudinal study

**DOI:** 10.7448/IAS.17.1.18585

**Published:** 2014-03-19

**Authors:** Nicola J Christofides, Rachel K Jewkes, Kristin L Dunkle, Mzikazi Nduna, Nwabisa Jama Shai, Claire Sterk

**Affiliations:** 1 School of Public Health, Faculty of Health Sciences, University of Witwatersrand, Johannesburg, South Africa; 2 Gender & Health Research Unit, South African Medical Research Institute, Pretoria, South Africa; 3 Rollins School of Public Health, Emory University, Atlanta, GA, USA; 4 Department of Psychology, University of Witwatersrand, Johannesburg, South Africa

**Keywords:** HIV/AIDS, adolescent pregnancy, South Africa, longitudinal data, sexual risk behaviour

## Abstract

**Introduction:**

Adolescents having unprotected heterosexual intercourse are at risk of HIV infection and unwanted pregnancy. However, there is little evidence to indicate whether pregnancy in early adolescence increases the risk of subsequent HIV infection. In this paper, we tested the hypothesis that adolescent pregnancy (aged 15 or younger) increases the risk of incident HIV infection in young South African women.

**Methods:**

We assessed 1099 HIV-negative women, aged 15–26 years, who were volunteer participants in a cluster-randomized, controlled HIV prevention trial in the predominantly rural Eastern Cape province of South Africa. All of these young women had at least one additional HIV test over two years of follow-up. Outcomes were HIV incidence rates per 100 person years and HIV incidence rate ratios (IRRs) estimated by Poisson multivariate models. Three pregnancy categories were created for the Poisson model: early adolescent pregnancy (a first pregnancy at age 15 years or younger); later adolescent pregnancy (a first pregnancy at age 16 to 19 years); and women who did not report an adolescent pregnancy. Models were adjusted for study design, age, education, time since first sexual experience, socio-economic status, childhood trauma and herpes simplex virus type 2 infection.

**Results:**

HIV incidence rates were 6.0 per 100 person years over two years of follow-up. The adjusted IRR was 3.02 (95% CI 1.50–6.09) for a pregnancy occurring at age 15 or younger. Women with pregnancies occurring between 16 and 19 years of age did not have a higher incidence of HIV (IRR 1.08; 95% CI 0.64–1.84). Early adolescent pregnancies were associated with higher partner numbers and a greater age difference with partners.

**Conclusions:**

Early adolescent pregnancies increase the incidence of HIV among South African women. The higher risk is associated with sexual risk behaviours such as higher partner numbers and a greater age difference with partners rather than a biological explanation of hormonal changes during pregnancy.

## Introduction

Adolescents having unprotected heterosexual intercourse are at risk of HIV infection and unwanted pregnancy. Several observational studies from South Africa have reported that the incidence of HIV is higher among pregnant women than among the general population [1, 2]. A prospective study conducted in Uganda found that HIV acquisition was higher among pregnant women than among either lactating women or non-pregnant and non-lactating women [3]. This study followed women over the immediate post-partum period (up to one year) and controlled for condom use and partner numbers. The authors conclude that the higher incidence among pregnant women is unlikely to be the result of sexual risk behaviours, and they attribute their finding to hormonal changes that affect immune responses or the genital tract mucosa [3]. Further investigation on pregnancies among young adolescents and over a longer follow-up period beyond the post-partum stage would add to our understanding of this phenomenon. There is also a need to better understand possible behavioural risk factors that may increase the risk of HIV acquisition among women who have an early adolescent pregnancy. Early adolescent pregnancy is defined in the literature as occurring at age 15 or younger [4]. A better understanding of early adolescent pregnancy and subsequent HIV infection would have important implications for prevention programs.

Incidences of HIV and pregnancy among adolescents remain high in South Africa. HIV prevalence among young women aged 15–19 is 6.9% and is 21.1% in the age range of 20–24 [5]. The adolescent fertility rate (childbirth at age 15–19) was 65 births per 1000 women [6, 7]. The 2003 Demographic Health Survey reported that 27.3% of women had a pregnancy while they were adolescents [8]. Most adolescents characterized their pregnancies as unplanned [9]. Pregnancies in adolescence have been associated with a range of short- and longer-term health and social consequences such as anaemia, urinary tract infection, pregnancy-induced hypertension, depression, substance abuse, increased sexual risk behaviour, as well as lower educational attainment and socio-economic status [10–20]. However, HIV infection has yet to be investigated as a possible health consequence of early adolescent pregnancy.

It is against this background that this study aimed to investigate the hypothesis that early adolescent pregnancy predicts incident HIV infections over two years of follow-up. This unique longitudinal data set allows us investigate this research question and explore the possible behavioural pathways through which this relationship may operate.

## Methods

### Participants

This study is based on results obtained from 1416 young South African women who were volunteer participants in a cluster-randomized, controlled trial through Stepping Stones, an HIV prevention intervention program. The young women, aged 15 to 26, were recruited from schools in 70 villages near Mthatha in the Eastern Cape, South Africa. Volunteers were eligible for enrolment if they were normally residents in the village where they were at school and if they were mature enough to understand the study and the consent process. All participants gave written informed consent. The 70 clusters were grouped into seven geographically defined strata. Within each stratum, equal numbers of clusters were randomly allocated to intervention or control. Women clustered in the intervention condition were randomized to receive a 17-session (50-hour) group intervention over a period of 3–12 weeks. Individuals in the control arm communities attended a single session of about three hours on HIV and safer sex.

The cohort was maintained using detailed contact information obtained at enrolment. Follow-up was undertaken nationwide to trace young people who had migrated away from the study area over the period of the study; 1099 women were successfully traced and provided data for the HIV incidence analyses. Detailed information about all assessments, study recruitment, access and ethical issues, including support for participants testing HIV positive, is published elsewhere [21]. Ethical clearance for the study was granted by the University of Pretoria ethics committee and the Emory University institutional review board. Written consent was obtained when participants were recruited into the study.

Assessments at baseline, 12 months and 24 months consisted of blood tests for HIV and herpes simplex virus type 2 (HSV2) and an interview to ascertain socio-demographic and partner characteristics and sexual risk behaviour. All questionnaires were administered by trained female interviewers. We used data to assess the effects of early (age 15 and younger) and later (age 16–19) adolescent pregnancies on incidence of HIV infection at two years of follow-up. For this longitudinal analysis, we excluded women who had HIV infection at baseline, women with missing data, and those who were lost to follow-up at 12 months and 24 months.

### Laboratory methods

HIV serostatus at baseline was assessed by the use of two rapid tests. The Determine (Abbott Diagnostics, Johannesburg, South Africa) test was used for screening, and samples with positive results were retested with Uni-Gold (Trinity Biotech, Dublin, Ireland). Indeterminate results were clarified by use of an HIV-1 screen enzyme-linked immunosorbent assay (ELISA) (Genscreen, Bio-Rad, Steenvoorde, France) followed by two confirmatory ELISAs if the sample was positive for HIV (Vironostika, BioMerieux, Marcy l'Etoile, France, and Murex 1.2.0, Murex Biotech, Dartford, UK). Towards the end of the second round of interviews, collection of blood as dried spots was introduced for some participants to ease logistics and to improve acceptability. In the third round of interviews, most blood was obtained as dried spots. The samples were tested with a screen and confirmatory ELISA. In this analysis, 745 (68%) of the final HIV outcomes were from dried blood spots equally distributed among participants who remained HIV-negative (*n*=658, 68%) and those who seroconverted (*n*=87, 68%). A glycoprotein G-based HSV2 ELISA was used to test for herpes infection (Kalon, Kalon Biological, Aldershot, UK). A CAPTIA HSV (Trinity Biotech) immunoglobulin G (IgG) type-specific ELISA was used to resolve discrepant results.

### Questionnaire

Detailed data were collected from all participants on socio-demographic characteristics, sexual behaviour and pregnancy history at each of three time points. The exposure of interest was a categorical variable based on age at first pregnancy, measured at the baseline assessment. Women were asked if they had ever been pregnant and, if so, in which year they first became pregnant. Age at first pregnancy was calculated by subtracting the date of birth from the date of first pregnancy. Three categories were created: early adolescent pregnancy, which included young women who experienced a first pregnancy at age 15 years or younger; later adolescent pregnancy, which included those who experienced their first pregnancy at age 16 to 19 years; and the referent group consisted of women who did not report an adolescent pregnancy.

Socio-demographic characteristics included age and years of schooling completed. Educational attainment was dichotomized into those who had completed more than 10 years of schooling and those who had 10 years of schooling or less at baseline. Socio-economic status was assessed by the use of a scale that encompassed household goods ownership, food and cash scarcity. Items on sexual partners included the age of the most recent partner. Age difference with main partner was calculated by subtracting the age of the participant from her partner's age. The age difference was dichotomized into an age difference of less than four years or four years or more.

A modified version of the short form of the Childhood Trauma Questionnaire was used [21, 22]. It included five dimensions of trauma: emotional neglect, physical neglect, emotional abuse, physical abuse and sexual abuse. Participants were asked whether—before the age of 18—they had experienced each act never, sometimes, often, or very often. Each dimension of adversity was then categorized as a three-level variable: the “never” exposure category required no exposure to any item in the dimension, the “some” exposure category was used when a participant responded “sometimes” to one item only, and an “often” exposure category was based on a response of “sometimes” to more than one item or any response of “often” or “very often.” The subscales were used separately in the analysis. The Cronbach's alpha for the scale was 0.77.

Items on sexual behaviour included partner numbers, condom use and age at first sex. Three questions established past year partner numbers of main boyfriends, *khwapheni* (hidden partners concurrent with main partners), and men with whom the participant had sex only once [21]. Condom use was measured by an item that asked participants whether they always, sometimes, seldom, or never used a condom. Transactional sex with a casual partner was measured based on questions asking about sex motivated by expectations of receiving one of a range of items [23].

Experience of intimate partner violence was measured by the World Health Organization (WHO) violence against women instrument [24]. The instrument was modified to be culturally appropriate. The instrument included five items measuring single and multiple occurrences of physical abuse occurring within the last 12 months and over a woman's lifetime, and four items measuring single and multiple occurrences of sexual abuse within the past 12 months and over a woman's lifetime.

### Statistical analysis

Because the original study was a stratified, two-stage survey with villages sampled from predefined strata based on geographical characteristics and participants clustered within villages, initial data analyses were carried out in Stata 10 using the survey procedures (Stata Corp., College Station, Texas, USA). These procedures allowed us to account for the lack of independence in the observations (non-zero, positive intra-cluster correlation (ICC)) because of the sampling design.

Descriptive statistics were first calculated for all variables; and two-way associations were determined between incident HIV infection and early and later adolescent pregnancy, childhood trauma, age at first sex, HSV, educational attainment, age and socio-economic status.

For each participant, we calculated the person years of exposure as the time from baseline to the last negative HIV result if the person remained negative, or as the total time between any negative tests as well as half the time between the last negative and first positive HIV test results. Random effects Poisson models were built to test the hypothesis that adolescent pregnancies occurring at the age of 15 years or younger, or between 16 and 19 years of age, predicted incident HIV infection measured at follow-up. Each model included variables for participation in the Stepping Stones study treatment arm, stratum and person years of exposure. We assessed the models for confounding by age, socio-economic status, education, child sexual, physical and emotional abuse, childhood emotional neglect, HSV status at baseline and age at first sex. Any variable found to affect the point estimate for the main exposure of interest by more than 10% was included in the final model [25]. We tested goodness of fit by using the Poisson test. We confirmed the findings of associations for the outcome variable by modelling survival time under observation with a Weibull model, with the same set of other variables included. To investigate whether results were robust to missing data, we undertook a sensitivity analysis with inverse probability weighting. The results suggest that the potential impact of missing data is minimal.

## Results

Of the 1415 women who were enrolled in the trial, 316 were excluded from this analysis. Women were excluded if they had HIV infection at baseline (*N*=159), missing data (*N*=1), or were lost to follow-up at 12 and 24 months (*N*=156). The 1099 women included in this analysis represent 88% of 1256 HIV-negative women in the trial.

Women lost to follow-up (Table 1) were older and were more likely to have had a boyfriend and sex at baseline. The mean age of the young women retained in the cohort was 17.5 years (15.2–18.9). At baseline, 87.3% of participants had had sexual intercourse. By the end of the approximately two years of follow-up, 93.6% of the young women had had sexual intercourse. Fifty-two young women had no sexual intercourse before the end of the study period. The median time between early adolescent pregnancy and the baseline assessment was six years with a range from three to eleven years.

**Table 1:** Socio-demographic and behavioural characteristics of HIV-negative women who were followed up and lost to follow-up

	Followed up (*N*=1099)	Lost to follow-up (*N*=156)	*p*
Age	18.40 (18.24 to 18.56)	18.78 (18.50 to 19.06)	0.01
Education (to grade 10)	956 (87%)	133 (85%)	0.59
Socio-economic status	0.0055 (−0.12 to 0.13)	−0.014 (−0.221 to 0.194)	0.85
Ever had a boyfriend	1059 (96%)	155 (99%)	0.05
Ever had sex	980 (89%)	148 (95%)	0.04
Ever had a pregnancy	197 (18%)	29 (19%)	0.86
Herpes simplex type 2 virus infection at baseline	245 (22%)	39 (26%)	0.37

The HIV incidence among the cohort of young women was 6.0 per 100 person years (*N*=128). As shown in Table 2, there were no significant differences in age, education, or socio-economic status between those women who acquired HIV and those who did not. Young women who experienced childhood adversity, particularly sexual and emotional abuse, were more likely to acquire HIV. A significantly greater proportion of women who acquired HIV, compared to those who did not, tested positive for HSV2 at baseline. Women who acquired HIV were younger when they first had sexual intercourse (15.3 years vs. 15.83; *p*=0.01) and were more likely to report an early adolescent pregnancy compared to those who did not seroconvert. Two hundred and fifty-one young women (17.74%) reported that they experienced a pregnancy when they were under the age of 19. Of these, 43 were aged 15 or younger when they had a pregnancy.

**Table 2:** Socio-demographic and behavioural characteristics of women associated with HIV incidence over two years of follow-up

	Incident HIV		No incident HIV		
			
	*N* (%) or mean	95% CI	*N* (%) or mean	95% CI	*p*
Age (years)	18.51	18.24–18.79	18.38	18.28–18.48	0.38
Education to grade 10	109/128 (87.24)	81.79–91.24	847/971 (85.04)	75.44–91.32	0.51
Socioeconomic status (SES)	0.001	−0.23–0.23	0.009	−0.08–0.09	0.95
Age at first sex	15.30	14.95–15.66	15.85	15.74–15.96	0.001
Childhood trauma					
Child sexual abuse					
None	68/684 (53.54)	44.07–62.76	616/684 (63.37)	59.87–66.74	0.05
Some	34/257 (26.77)	20.44–34.22	223/257 (22.94)	20.46–25.63	
Often	25/158 (19.69)	13.73–27.40	133/158 (13.68)	11.65–16.0	
Emotional abuse					
None	41/496 (32.28)	23.92–41.96	455/496 (46.81)	43.14–50.52	<0.01
Some	49/365 (38.58)	30.91–46.87	316/365 (32.51)	29.82–35.32	
Often	37/238 (29.13)	22.41–36.92	201/238 (20.68)	17.99–23.65	
Physical abuse					
None	8/124 (6.3)	10.19–13.93	116/124 (11.93)	3.21–11.98	0.08
Some	16/171 (12.6)	13.44–18.81	155/171 (15.95)	7.68–19.98	
Often	103/804 (81.1)	72.95–87.23	701/804 (72.12)	68.85–75.17	
Emotional neglect					
None	73/651 (57.94)	49.51–65.92	578/651 (59.47)	56.2–62.65	0.44
Some	33/247 (26.19)	19.55–34.13	214/247 (22.02)	19.49–24.77	
Often	20/200 (15.87)	10.84–22.65	180/200 (18.52)	15.99–21.35	
Adolescent pregnancy					
None	96/127 (75.59)	67.55–82.17	823/972 (84.67)	81.87–87.11	<0.001
≤15 years	10/127 (7.87)	4.11–14.55	21/972 (2.16)	1.41–3.3	
16–19 years	21/127 (16.54)	10.92–24.24	128/972 (13.17)	10.95–15.75	
HSV2 infection at baseline	52/128 (40.6)	31.3–50.0	193/971 (19.9)	17.2–19.6	<0.001

The incidence of HIV and incidence rate ratio (IRR) derived from the adjusted multivariable Poisson model is shown in Table 3. Women who had an early adolescent pregnancy, aged 15 years or younger, were three times more likely to acquire HIV (IRR=3.02; 95% confidence interval 1.50–6.09). Women who experienced a later adolescent pregnancy (aged 16–18 years) did not have an increased risk of incident HIV compared with the young women who did not have an adolescent pregnancy. In the model, we adjusted for childhood trauma, age at first sex, HSV2, study design, educational attainment, socio-economic status and age.

**Table 3:** Relative HIV incidence with exposure to early and later adolescent pregnancy

	IRR	*p*	95% confidence interval	
Pregnancy ≤15 years	3.02	<0.01	1.50	6.09
Pregnancy ≥16 and <19	1.08	0.77	0.64	1.84

Controlled for Stepping Stones study, age, socio-economic status, education, child sexual abuse, child emotional abuse, child physical abuse, emotional neglect, HSV, age at first sex.

Given the strong link between an early adolescent pregnancy and subsequent HIV infection, we explored whether adolescents who experienced an early pregnancy had an increased risk of a range of behavioural factors (Table 4) and whether the young women had partners with characteristics that placed them at a differential risk of acquiring HIV (Table 5). Women who had experienced an early adolescent pregnancy had increased odds of having four or more sexual partners in their lifetime. Although marginally significant, they also experienced more physical and sexual violence than women who had a later adolescent pregnancy or did not have a pregnancy. A greater proportion of women who had an early adolescent pregnancy reported always using a condom at the baseline assessment than those women who experienced a later adolescent pregnancy (25.8% vs. 14.0%). Women who had experienced an early adolescent pregnancy had partners at the start of the study who were much older (four or more years older). However, fewer of these women reported that their partners were in concurrent relationships than those women who had a later adolescent pregnancy.

**Table 4:** Early and later adolescent pregnancy and subsequent behavioural outcomes

Sexual risk behaviours:	No pregnancy (*N*=799)	95% CI	Pregnancy ≤15 (*n*=31)	95% CI	Pregnancy 16–19 (*N*=150)	95% CI	*p*
**Condom use**							*p*=0.01
Always	24.16	20.74–27.93	25.81	13.92–42.80	14.00	8.9–21.34	
	193/799		8/31		21/150		
Sometimes/often	30.66	26.66–34.99	29.03	17.55–44.01	30.67	23.35–39.10	
	245/799		9/31		46/150		
Never	45.18	40.27–50.19	45.16	31.37–59.74	55.33	46.97–63.41	
	361/799		14/31		83/150		
**Partner numbers**	*N*=812		*N*=31		*N*=151		*p*=0.01
1	68.97	65.60–72.15	64.52	47.85–78.28	72.19	63.23–79.66	
	560/812		20		109		
2 to 3	28.45	25.54–31.54	22.58	11.91–38.62	24.5	18.00–32.43	
	231		7		37		
4+	2.59	1.75–3.81	12.9	5.15–28.80	3.31	1.37–7.80	
	21		4		5		
**Type of Intimate Partner Violence (IPV)**	*N*=887		*N*=31		*N*=151		*p*=0.11
None	67.42	63.88–70.77	58.06	38.91–75.06	72.85	65.58–79.07	
	598/887		18/31		110/151		
Physical only	20.07	17.39–23.05	19.35	9.12–36.47	20.53	15.23–27.09	
	178/887		6/31		31/151		
Sexual	5.98	4.32–8.21	6.45	1.66–21.98	1.32	0.33–5.22	
	53/887		2/31		2/151		
Physical and sexual	6.54	4.95–8.59	16.13	6.18–35.95	5.3	2.39–11.32	
	58/887		5/31		8/151		
**Any transactional sex**	23.99	20.26–28.15	25.81	12.99–44.77	28.48	21.72–36.36	*p*=0.52
	195/813		8/31		43/151		
**Relationship control**	796		30		146		*p*=0.89
Low equity	33.17	29.18–37.41	30	16.24–48.65	34.93	27.10–43.67	
	264/796		9/30		51/146		
Medium equity	52.64	48.84–56.40	50	30.84–69.16	52.05	43.27–60.71	
	419/796		15/30		76/146		
High equity	14.2	11.48–17.43	20	8.44–40.40	13.01	8.33–19.75	
	113/796		6/30		19/146		

Note: Teenage pregnancy in this exploratory analysis is the exposure of interest and the sexual risk behaviours are the outcomes.

**Table 5:** Partner characteristics at baseline of women who had an early adolescent pregnancy

	No pregnancy (*N*=869)	95% CI	Pregnancy ≤15 (*n*=30)	95% CI	Pregnancy ≥16 and ≤19 (*N*=146)	95% CI	*p*
Age difference of 4+ years	31.21	27.87–34.77	63.33	43.26–79.65	49.32	40.62–58.06	*p*=0.000
	270/865		19/30		72/146		
Partner probably or definitely has another partner	63.52	59.78–67.11	66.67	49.71–80.18	78.08	70.49–84.16	*p*=0.003
	552/869		20/30		114/146		

## Discussion

Results support the hypothesis that young women who have an early, but not later, adolescent pregnancy are more likely to subsequently become HIV infected. The study provides strong evidence of the temporal aspect of this finding (with pregnancies occurring years before the incident HIV infection) thus ruling out the possibility that HIV infection occurred simultaneously or preceded the early pregnancies. This finding suggests that behavioural factors may be important in the increased risk of incident HIV, adding to the results of earlier studies that suggest that higher transmission in pregnancy is biological and the result of hormonal changes during pregnancy [3].

Early adolescent pregnancy was associated with higher lifetime partner numbers and subsequently having a partner who was four or more years older. Although this analysis was exploratory, these findings suggest that early adolescent pregnancies were followed by different risk behaviour than that among young women who had a pregnancy between the ages of 16 and 18 years and the younger adolescents. Further research is required to investigate the pathways through which early adolescent pregnancy increases the risk of subsequent HIV infection.

This paper builds on previous work on this data set. Jewkes and colleagues found that child sexual abuse increased the risk of subsequent HIV infection among the same group of young women [26]. In our analysis, we adjusted for different dimensions of childhood trauma. Adolescents reporting an early pregnancy at the age of 15 or younger would all, by legal definition, have experienced child sexual abuse. However, qualitative data from the participants in this study indicated that, although some of these early relationships were experienced as abusive, others were described as more equitable [27]. There is a complex interplay between adolescents’ perceptions of autonomy and ability to consent to sexual intercourse, and the need for protective measures to prevent child sexual abuse.

Behavioural interventions are common that address adolescent sexual risk behaviour and that aim to reduce unwanted pregnancies and HIV infection [28–32]. Some studies have investigated adolescent pregnancy and HIV as co-occurring outcomes, and prevention interventions have focused on the simultaneous prevention of both [33, 34]. This study suggests the importance of preventing early adolescent pregnancy as related to subsequent HIV infection. Interventions that focus on gender equality and sexual rights (particularly on the delay of first sexual intercourse) are critical.

The South African Children's Act 38 of 2005 ensures confidentiality for young women under the age of 18 who obtain condoms, contraceptives, or contraceptive advice. Despite the law, adolescents report experiencing judgemental attitudes from health care providers when they access contraceptive services [35]. They also are afraid that health care providers will not maintain confidentiality and will discuss their contraceptive use with parents or relatives. Another paper from this data set found that very few adolescents who reported having had sexual intercourse accessed contraceptives prior to having an adolescent pregnancy and that most contraceptive use by adolescents followed a pregnancy [36].

This study has several limitations. It is based on the analysis of data from a cohort of volunteers in an HIV prevention trial, and this may limit the generalizability of the findings. Although retention of study participants was high, some were lost to follow-up, and there were a few significant differences in the socio-demographic characteristics. We tested for robustness to missing data, and our results suggested that the potential effect was small. Pregnancies were self-reported, and it is possible that pregnancies that were terminated or miscarried were under-reported. Significant strengths of the study include the use of prospectively collected data, HIV testing protocol, and the coherence and strength of our findings across different modelling procedures.

## Conclusions

This study found that women who experienced an early adolescent pregnancy had an increased incidence of HIV infection that occurred two or more years afterwards. Early adolescent pregnancies may lead to sexual risk behaviours such as higher partner numbers and a greater age difference with partners rather than only a biological explanation of hormonal changes during pregnancy. There is a need to address adolescent pregnancy not only as a health outcome but as a risk factor for HIV infection. Thus, preventing early adolescent pregnancies is important in a comprehensive HIV prevention strategy in countries with high HIV prevalence, such as South Africa.
